# The cost of diabetes chronic complications among Iranian people with type 2 diabetes mellitus

**DOI:** 10.1186/2251-6581-13-42

**Published:** 2014-03-04

**Authors:** Amir Farshchi, Alireza Esteghamati, Ali Akbari Sari, Abbas Kebriaeezadeh, Mohammad Abdollahi, Farid Abedin Dorkoosh, Mohamad Ebrahim Khamseh, Rokhsareh Aghili, Abbas Keshtkar, Maryam Ebadi

**Affiliations:** 1 Department of Pharmacoeconomics and Pharmaceutical Administration, School of Pharmacy, Tehran University of Medical Sciences, Tehran, Iran; 2 Endocrinology and Metabolism Research Center (EMRC), Vali-Asr Hospital, Tehran University of Medical Sciences, Tehran, Iran; 3 Department of Health Management and Economics, Tehran University of Medical Sciences, Tehran, Iran; 4 Department of Toxicology and Pharmacology, School of Pharmacy and Pharmaceutical Sciences Research Center, Tehran University of medical Sciences, Tehran, Iran; 5 Endocrine Research Center, Institute of Endocrinology and Metabolism, Iran University of Medical Sciences, Tehran, Iran; 6 Osteoporosis Research Center, Endocrinology and Metabolism Research Institute, Tehran University of Medical Sciences, Tehran, Iran

**Keywords:** Cost, Type 2 diabetes mellitus, Complication, HbA1c, Productivity, QALY

## Abstract

**Background:**

To evaluate the cost of diabetes related micro- and macrovascular complications in Iranian people with type 2 diabetes mellitus.

**Methods:**

In routine clinical practice, people with type 2 diabetes mellitus were assessed for 10 years at a diabetes care center. The type of medications and clinical data were extracted from patients’ documents. Mortality rate and the incidence of micro- and macrovascular complications recorded in patients’ documents were analyzed. Cost analysis was comprised of 1) para clinic costs as well as laboratory, medications, clinical visits and nonmedical costs 2) inpatient costs as well as hospital admission costs, disability, and mortality costs.

**Results:**

From 1562 people with type 2 diabetes mellitus, a total of 1000 patients with mean duration disease of 11.2 years, who had completed information in their documents, were studied. All people were free from complications at baseline. Mean cumulative incidence of diabetes-related complications over 10 years were 10.9 ± 3.5%, 8.0 ± 3.1%, 4.6 ± 1.7%, 9.1 ± 3.6% and 2.3 ± 0.9% for peripheral neuropathy and diabetic foot ulcer, nephropathy, ophthalmic complications, cardiovascular disease and death, respectively. People with better glycemic control had less complication and also related expenditures. Average para clinic cost per patient was 393.6 ± 47.8 and average inpatient cost per patient was 1520.7 ± 104.5 USD.

**Conclusions:**

Our findings demonstrate considerable incidence of diabetes chronic complications and also high health care expenditure for related complications among our patients. As the number of people with diabetes continues to rise, early detection of the disease and implementation of timely and appropriate therapeutic strategies could decrease the burden of diabetes chronic complications and also huge related expenditures.

## Background

The prevalence of type 2 diabetes mellitus is rising dramatically worldwide, mostly in developing countries. It is associated with highly cost-demanding complications [1-3]. In 2011, the number of people with diabetes mellitus was estimated to be 366 million. Present continuous trend would lead to 10% of the world population with diabetes by 2030 [4]. The data represented by the National Survey of Risk Factors for Non-Communicable Diseases of Iran indicate that 7.7% of adults younger than 65 years had type 2 diabetes in 2008 [1]. Glycemic control is the main step of treatment in people with type 2 diabetes and could be reached via nutritional therapy [5,6], physical activity, oral glucose lowering drugs (OGLDs), and insulin therapy [7,8]. Poor glycemic control results in chronic complications including microvascular (peripheral neuropathy and foot ulcer, nephropathy and retinopathy) and macrovascular diseases (cardiovascular, cerebral and peripheral vascular disease) [9,10] with profound impact on the quality of life of millions of patients and their families [11]. In addition, diabetes as one of the most cost-demanding health conditions imposes a large economic burden to the healthcare system, most of which is the monetary value associated with disability and loss of life as a result of the disease itself and its related complications [12]. Oglesby et al. reported that diabetes related costs were 16% and 20% lower in people with good glycemic control compared with those with fair glycemic control and poor glycemic control [13]. Menzin et al. found that people with a mean A1C ≥ 10% had higher diabetes related hospital costs than those with a mean A1C <7% [14]. It has been estimated that global health expenditures of diabetes and its complications were about 376 billion US dollar (USD) in 2010, while a rise to 490 billion USD in 2030 is anticipated [15]. Although the results from previous studies have assumed costs, disability and loss of life associated with the disease in people with diabetes [16,17], to the best of our knowledge, there is not any report on incidence of diabetes chronic complications and concomitant costs in the routine clinic practice in Iran. So, the incidence and cost of diabetes chronic complications in Iran are still under debate.

The aim of this study was to evaluate the incidence of micro- and macrovascular complications as well as to analyze the costs of complications in people with type 2 diabetes in the routine clinical practice.

## Methods

In a routine clinical practice people with type 2 diabetes (ICD-9/diagnostic code 250.x) were assessed retrospectively for 10 years from 1 December 2002 to 1 December 2012 at a public diabetes care center using paper based medical records. This center cares for over 1000 diabetic patients per month. People who had at least one follow-up visit per year were included. Demographic characteristics were recorded at the beginning of study. The type of the antidiabetic medications administered and clinical data, including hemoglobin A1C (HbA1c), fasting blood glucose (FBG), total cholesterol (TC), high-density lipoprotein cholesterol (HDL-C), low-density lipoprotein cholesterol (LDL-C), triglyceride (TG), body mass index (BMI) and urine micro-albumin were extracted from patients’ documents yearly. Mortality rate and incidence of micro- and macrovascular complications recorded in patients’ documents were also reported. Diabetes chronic complications were defined as peripheral neuropathy and diabetic foot ulcer (ICD-9/diagnostic codes 250.6, 707, 736), nephropathy (ICD-9/diagnostic codes 580–586), ophthalmic complications (ICD-9/diagnostic codes 362.01, 365, 366) and cardiovascular disease (CVD) (ICD-9/diagnostic codes 401–405,410-413,414.0–414.8) (Table 1).

**Table 1:** ICD-9/diagnostic codes for type 2 diabetes chronic complications

**Code**	**Disease**
**- Cardiovascular disease**	
401	Essential hypertension
402	Hypertensive heart disease
403	Hypertensive renal disease
404	Hypertensive heart and renal disease
405	Secondary hypertension
410	Acute myocardial infarction
411	Other acute and sub-acute forms of ischemic heart disease
412	Old myocardial infarction
413	Angina pectoris
414.0	Coronary atherosclerosis
414.1	Aneurysm and dissection of heart
414.8	Ischemic heart disease, chronic heart disease.
**- Peripheral neuropathy and foot ulcer**	
250.6	Diabetes with neurological manifestations
707	Chronic ulcer of skin
736	Other acquired deformities of limbs
**- Nephropathy**	
580	Acute glomerulonephritis
581	Nephrotic syndrome
582	Chronic glomerulonephritis
583	Nephritis and nephropathy, not specified as acute or chronic
584	Acute renal failure
585	Chronic renal failure
586	Renal failure, unspecified
**- Ophthalmic complications**	
362.01	Background diabetic retinopathy, diabeticmacular edema
362.2	Proliferativeretinopathy
365	Glaucoma
366	Cataract

Patients were screened annually for peripheral neuropathy using history of pinprick sensation and tests such as vibration perception (using a 128-Hz tuning fork) and monofilament pressure sensation. Foot ulcer was also screened by medical examination annually. Urine albumin excretion test was assessed annually for diabetes nephropathy. The serum creatinine was used to estimate glomerular filtration rate (GFR) and to assess the stage of chronic kidney disease (CKD). Additional assessment was considered by a nephrologist if needed. Dilated and comprehensive eye examination was done by an ophthalmologist annually. Typical or atypical cardiac symptoms were asked and resting electrocardiography (ECG) was recorded annually. If there was any abnormality in history and resting ECG, people were referred for further assessments by a cardiologist. All additional notes regarding diabetes chronic complications in patients’ documents were also analyzed and the first unmoral findings in mentioned tests were considered as the incidence of complication. Ethical approval was granted from the ethics board at Tehran University of Medical Sciences.

### Laboratory measurements

After an overnight fasting of at least 12 hours, venous blood samples were drawn and were sent to hospital laboratory for analysis. All samples were analyzed by unique laboratory center. FBG concentrations were determined using the glucose oxidase method. Percentage of HbA1c was measured using the high performance liquid chromatography (HPLC) method [18]. Serum concentrations of HDL, LDL and TG were determined using enzymatic methods with available commercial kits (Pars Azmun, Karaj, Iran) in a Hitachi 704 automatic analyzer (Tokyo, Japan) [19].

### Costs

#### Direct medical costs

Medical costs of each patient were obtained by a checklist from their documents, described in Table 2. Any pharmaceutical, laboratory/diagnostic and rehabilitative care as well as visiting specialists, general practitioners, nurses, opticians, podiatrists, and dieticians as well as any hospital admissions were recorded annually. All related costs were calculated using governmental prices and finally total costs were adjusted to 2012 USD.

**Table 2:** Average direct medical cost for type 2 chronic diabetes complications (USD)

**Diabetes chronic complications**	**Gender**		**Age**			**Glycemic control (% A1C)**		
	**Male**	**Female**	**<40**	**40-60**	**>60**	**<7**	**7-8.5**	**>8.5**
**Neuropathy & diabetic foot ulcer**								
Additional visits (Infectious disease specialist, Surgeon, …)	5.2 ± 1.2	6.1 ± 2.2	6.3 ± 2.3	6.8 ± 3.3	6.9 ± 2.7	2.5 ± 1.1	5.6 ± 2.2	12.4 ± 5.9
Medications (Gabapentin, Pregabalin, Antibiotics, …)	3.2 ± 1.1	2.4 ± 1.1	3.0 ± 1.5	3.3 ± 1.2	3.3 ± 1.6	0.5 ± 0.1	2.7 ± 1.5	6.5 ± 3.2
Laboratory tests	6.1 ± 2.2	5.5 ± 2.4	7.0 ± 4.2	7.6 ± 4.3	7.7 ± 3.7	3.6 ± 1.2	5.8 ± 3.1	12.7 ± 6.8
Nerve conduction velocity (NCV)	8.5 ± 3.1	4.7 ± 2.6	9.6 ± 4.7	10.4 ± 4.9	10.5 ± 4.1	5.7 ± 2.4	10.3 ± 6.1	14.5 ± 7.4
Laser therapy	1.9 ± 0.9	1.1 ± 0.5	0.6 ± 0.1	1.4 ± 0.3	3.0 ± 0.9	0.7 ± 0.1	1.5 ± 0.5	2.9 ± 0.9
Shoes	1.2 ± 0.4	1.3 ± 0.7	1.4 ± 0.4	1.5 ± 1.1	1.5 ± 0.5	0.8 ± 0.3	1.1 ± 0.5	2.3 ± 1.6
Foot scan	1.4 ± 0.7	2.0 ± 0.9	1.8 ± 0.7	2.0 ± 1.2	2.0 ± 0.8	0.4 ± 0.2	2.1 ± 0.7	3.4 ± 1.5
Dressing and wound care	1.6 ± 0.4	2.4 ± 1.1	2.2 ± 0.8	2.3 ± 1.3	2.4 ± 1.1	0.6 ± 0.4	2.1 ± 0.6	4.3 ± 2.4
Amputation	21.3 ± 7.8	17.8 ± 6.5	14.3 ± 6.6	12.3 ± 8.1	12.4 ± 7.2	5.4 ± 2.4	13.2 ± 5.8	20.5 ± 10.8
Prosthesis	12.1 ± 3.9	8.8 ± 4.6	13.9 ± 4.9	15.1 ± 6.3	15.3 ± 6.6	6.5 ± 3.5	13.9 ± 7.4	24.1 ± 12.1
Bed day	23.1 ± 5.9	19.5 ± 9.4	19.3 ± 7.2	26.0 ± 10.1	31.1 ± 9.2	12.2 ± 4.9	23.1 ± 8.5	37.5 ± 13.2
**Total**	**85.6 ± 4.6**	**71.6 ± 6.2**	**79.4 ± 7.1**	**88.6 ± 8.8**	**96.1 ± 6.9**	**38.9 ± 10.9**	**81.4 ± 11.7**	**141.13 ± 23.9**
**Nephropathy**								
Additional visits (Nephrologist, …)	14.4 ± 6.6	11.0 ± 4.3	10.6 ± 4.6	11.7 ± 5.1	24.0 ± 3.9	6.7 ± 3.7	9.2 ± 3.6	20.1 ± 7.9
Medications	11.0 ± 5.9	8.4 ± 3.3	8.1 ± 3.8	8.9 ± 4.4	20.7 ± 4.3	4.2 ± 1.9	9.3 ± 4.3	14.2 ± 4.9
Laboratory tests (24 hr. UA)	11.3 ± 5.3	8.7 ± 2.9	8.3 ± 3.5	9.2 ± 3.7	21.0 ± 4.4	3.2 ± 1.4	8.4 ± 2.6	16.6 ± 5.3
Dialysis	70.5 ± 20.1	53.8 ± 18.8	51.8 ± 15.3	57.1 ± 25.5	118.4 ± 29.8	23.6 ± 9.8	56.5 ± 21.1	97.5 ± 33.2
Kidney transplantation	48.5 ± 19.8	37.0 ± 15.6	35.7 ± 14.7	39.3 ± 19.4	67.1 ± 20.2	20.4 ± 8.3	41.7 ± 20.2	60.2 ± 27.6
Bed day	9.5 ± 4.2	7.2 ± 4.1	7.0 ± 2.8	7.7 ± 3.3	9.2 ± 6.2	2.9 ± 1.1	6.4 ± 3.1	14.5 ± 7.9
**Total**	**165.3 ± 14.4**	**126.1 ± 9.7**	**121.4 ± 15.5**	**133.9 ± 20.1**	**260.3 ± 12.5**	**61.0 ± 20.2**	**131.5 ± 55.8**	**223.1 ± 89.9**
**Retinopathy**								
Additional visits (Ophthalmologist, …)	9.4 ± 3.3	8.5 ± 2.3	8.2 ± 4.1	8.9 ± 4.5	9.9 ± 4.8	3.7 ± 1.2	8.9 ± 4.4	14.8 ± 6.8
Medications (Bevacizumab, …)	13.4 ± 6.6	12.0 ± 5.8	11.6 ± 5.4	12.7 ± 8.8	14.0 ± 7.7	5.6 ± 2.3	11.8 ± 5.3	17.3 ± 8.5
Laboratory tests	11.8 ± 4.3	10.6 ± 4.9	10.2 ± 5.9	11.2 ± 5.7	12.4 ± 5.8	5.1 ± 2.5	12.1 ± 8.3	16.6 ± 5.9
Laser therapy	26.7 ± 9.7	24.0 ± 12.2	23.2 ± 12.8	25.3 ± 16.5	28.1 ± 17.4	11.1 ± 8.1	23.7 ± 11.9	40.1 ± 21.3
Surgery	14.0 ± 7.6	11.7 ± 5.5	9.4 ± 6.2	10.9 ± 5.9	13.9 ± 8.3	4.8 ± 2.8	12.6 ± 4.9	16.5 ± 8.4
Bed day	9.1 ± 4.8	8.7 ± 3.2	11.0 ± 6.1	11.8 ± 5.4	14.2 ± 8.5	5.9 ± 2.9	12.3 ± 7.7	18.9 ± 8.4
**Total**	**84.4 ± 4.9**	**75.8 ± 6.3**	**73.2 ± 3.1**	**79.8 ± 11.3**	**88.8 ± 6.6**	**36.2 ± 18.5**	**81.4 ± 32.8**	**124.2 ± 48.3**
**Cardiovascular disease**								
Additional visits (Cardiologist, …)	3.8 ± 1.6	4.0 ± 1.9	3.5 ± 1.2	3.8 ± 1.2	4.2 ± 1.8	1.2 ± 0.5	3.5 ± 1.3	7.0 ± 3.2
Medications (Clopidogrel, Nitrocontin, …)	6.4 ± 1.8	6.7 ± 2.7	5.8 ± 2.1	6.3 ± 3.6	7.0 ± 4.1	3.3 ± 0.8	6.9 ± 2.3	9.4 ± 3.7
Echocardiography	16.2 ± 7.6	17.1 ± 8.4	14.8 ± 6.5	16.1 ± 6.9	27.7 ± 4.0	4.9 ± 1.1	15.9 ± 5.4	27.8 ± 14.6
Exercise test	2.4 ± 1.0	2.5 ± 1.1	2.1 ± 0.8	2.3 ± 0.7	2.6 ± 1.4	0.4 ± 0.1	1.9 ± 0.4	4.6 ± 3.1
CCU impatience	19.7 ± 8.6	20.7 ± 7.3	18.0 ± 6.4	19.6 ± 6.4	31.5 ± 7.7	7.5 ± 3.1	18.9 ± 8.9	32.1 ± 17.5
Doppler sonography (Foot, head and neck)	19.2 ± 7.8	20.2 ± 9.3	17.5 ± 9.7	19.0 ± 7.2	40.9 ± 8.5	6.9 ± 3.2	19.0 ± 7.3	31.1 ± 17.9
Angiography (Heart, foot)	16.4 ± 8.3	17.2 ± 9.1	14.9 ± 6.6	16.3 ± 5.9	27.8 ± 5.9	6.1 ± 2.6	15.0 ± 8.8	27.8 ± 16.3
Stent	40.8 ± 26.3	42.9 ± 20.5	37.2 ± 16.7	40.5 ± 20.0	94.5 ± 24.4	18.9 ± 5.9	35.6 ± 13.3	67.6 ± 23.8
Vascular surgery, coronary artery bypass graft	24.0 ± 9.9	25.2 ± 8.5	21.9 ± 7.4	23.8 ± 9.1	76.1 ± 10.5	13.3 ± 8.8	22.1 ± 12.7	36.7 ± 20.6
Heart transplantation	56.5 ± 26.8	59.4 ± 27.9	51.5 ± 25.6	56.1 ± 18.9	111.6 ± 12.9	23.7 ± 16.4	55.9 ± 34.8	90.5 ± 32.5
Bed day	8.9 ± 4.2	9.3 ± 5.4	8.1 ± 4.7	8.8 ± 5.5	9.7 ± 4.8	2.2 ± 0.3	8.9 ± 4.4	15.3 ± 7.3
**Total**	**214.1 ± 12.8**	225.3 ± 24.9^*^	**195.4 ± 19.5**	**212.7 ± 20.2**	433.5 ± 17.3*	**88.4 ± 20.8**	**203.6 ± 77.8**	349.9 ± 91.5**

*P < 0.05, **P < 0.01 related to F test.

#### Direct non-medical costs

A patient self-estimate questionnaire was used to assess non-medical expenditures due to services such as transportation for patients and their family to clinic and taking care of dependents.

#### Indirect costs

Costs of productivity loss due to diabetes -related health problems were determined annually by days absent from work, poor work performance, low earning's capacity from disabilities, and labor loss due to early mortality. Mortality costs were calculated as the lost earnings owing to premature mortality. We calculated the number of years of life lost and also the number of death in different age and gender group attributable to type 2 diabetes using our survival information. The number of days absent from work due to diabetes -related health care was recorded and the average of net daily wage was asked from each patient; then, the loss of value because of work absence was calculated using the formula below:

Value loss due to absences from work (USD) = Number of days absent from work × Average of daily wage (USD).

To retrieve their indemnity claims during one year we used their insurance ID from the social security organization (SSO) database. At the end, all costs were categorized in two groups; para-clinic and inpatient costs. Costs from the health society perspective were converted from Iranian Rials (IRR) into USD at an official exchange rate of 12,260 IRR/USD 2012 in order to facilitate international comparison [20]. Average annual inflation rates of 21.5% in health care expenditures and 18.6% in gross incomes from 2002 to 2012 were applied to the costs [20].

#### Statistical analysis

Data are presented as Mean ± standard deviation (S.D.). Incidences were calculated using the whole number of people who experienced new complications yearly as the numerator and the total number of people alive during the same period as the denominator. Only the first event in a given year contributes to each rate and cumulative incidence of each complication was calculated. Considering age, gender and diabetic control status, stratification analysis was conducted. Subgroup analysis for status of glycemic control was defined in three categories: good control (A1C <7%), fair control (7% < A1C <8.5%) and poor control (A1C >8.5%). The assumptions of normal distribution as well as the equality of variance in subgroups were evaluated and if the assumptions failed, transformations were used to induce normality. Categorical and continuous variables were compared using Chi-square and Student’s t test, respectively. Mixed between-within analysis of variance (ANOVA) and nonparametric tests were performed for between group comparisons with post-hoc followed test. P values < 0.05 were considered statistically significant.

## Results

**Figure 1:**
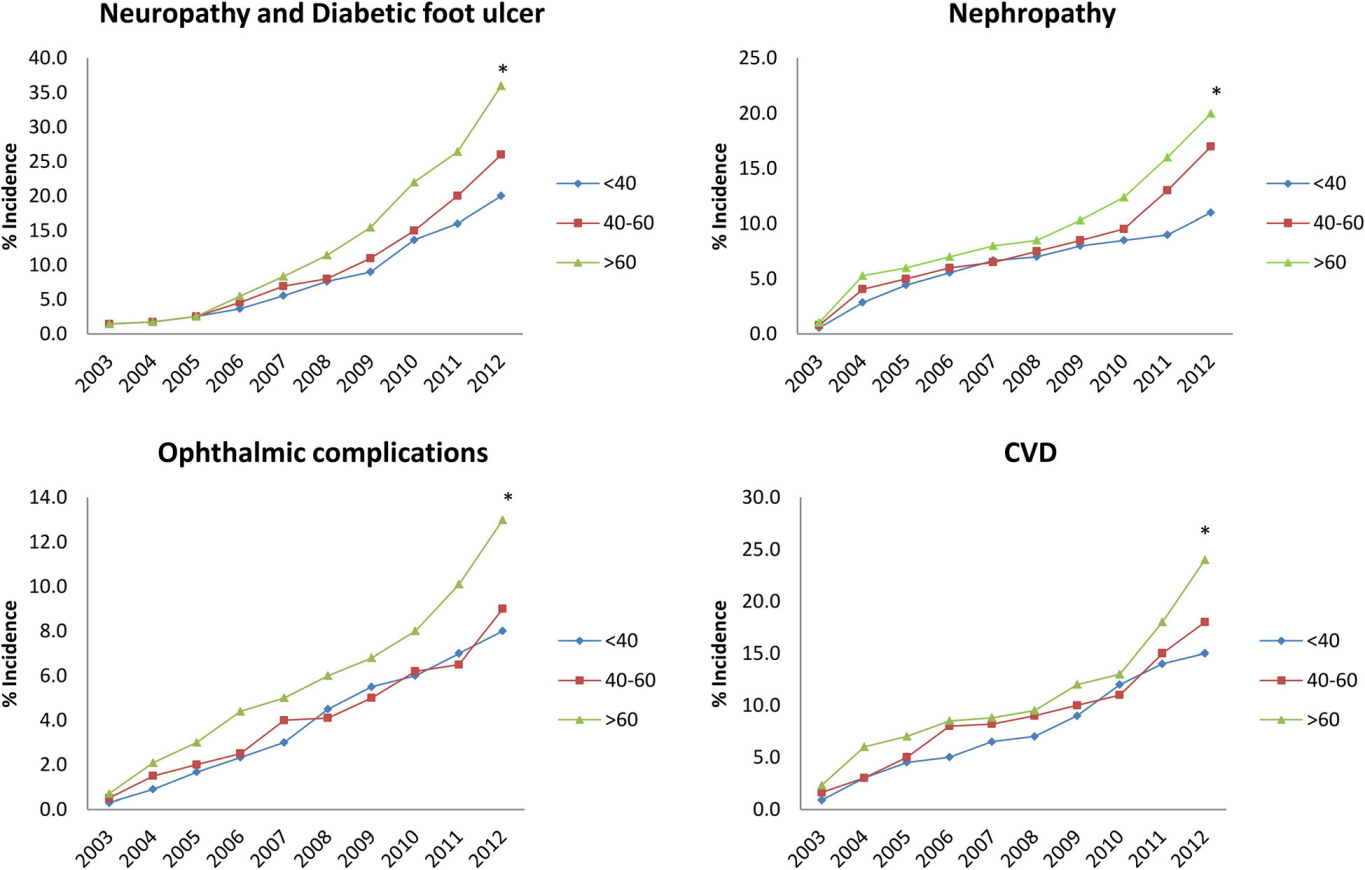
**Diabetes chronic complication incidences in age categorized during 2003 to 2012.** *P < 0.05 related to F test.

A total of 1562 people with type 2 diabetes from 1 December 2002 to 1 December 2012 were regularly referred to a clinical endocrinology and metabolism center in Tehran, Iran. Five hundred and sixty two people were excluded, and a total of 1000 people were assessed. The main exclusion criteria were incomplete documents, missed follow-up and gestational diabetes. Baseline characteristics and clinical parameters are shown in Table 3. There were no significant differences between men and women regarding baseline characteristics (P = 0.56). The mean age was 53.5 ± 9.4 years (M: 55.5 ± 8.1, F: 51.8 ± 7.6). Mean duration of diabetes was 11.2 ± 4.2 years in total population, whereas it was 4.7 ± 1.5, 9.2 ± 2.6 and 16.1 ± 5.5 in age category of <40, 40–60 and >60, respectively. After 10 years, clinical characteristics were improved (Table 3). Total diabetes related new events over 10 years were 289, 155, 103, 180 and 78 for peripheral neuropathy and diabetic foot ulcer, nephropathy, ophthalmic complications, CVD and death, respectively. So, yearly cumulative incidences of diabetes-related events over 10 years were 10.9 ± 3.5%, 8.0 ± 3.1%, 4.6 ± 1.7%, 9.1 ± 3.6% and 2.3 ± 0.9%, respectively (Table 4). All cumulative incidence rates were higher in people older than 60 years (Figure 1). A significant gender difference was also noted (P = 0.023), with men showing a higher incidence rate in diabetes chronic complications (Table 4).

**Table 3:** Characteristics of study population

**Baseline characteristics**											
**Parameters**		**Number (%)**	**Duration of diabetes (Year)**	HbA1c * (%)	FBG* (mg/dL)	TC* (mg/dL)	HDL-C* (mg/dL)	LDL-C* (mg/dL)	TG* (mg/dL)	BMI* (Kg/m^**2**^**)**	**Micro-albumin (mg/kg)**
**Gender**	**Male**	468 (46.8)	10.8 ± 6.1	8.8 ± 3.2	160.7 ± 28.7	175.9 ± 36.8	42.1 ± 11.4	99.2 ± 27.6	178.8 ± 34.4	28.16 ± 5.9	82.7 ± 21.7
	**Female**	532 (53.2)	11.7 ± 5.7	8.5 ± 2.8	163.5 ± 32.5	191.7 ± 45.2	48.2 ± 9.1	107.8 ± 30.1	190.9 ± 41.8	31.4 ± 6.9	67.3 ± 17.3
**Age**	**<40**	119 (11.9)	4.7 ± 1.5	7.8 ± 2.3	155.7 ± 48.3	192.3 ± 43.3	43.4 ± 10.3	109.32 ± 33.4	210.0 ± 51.4	30.8 ± 7.6	53.2 ± 10.8
	**40–60**	512 (51.2)	9.2 ± 2.6	8.6 ± 4.4	164.6 ± 44.8	186.1 ± 54.9	44.4 ± 11.1	104.76 ± 41.7	198.6 ± 53.4	29.5 ± 6.4	71.7 ± 16.7
	**>60**	369 (36.9)	16.1 ± 5.5	8.7 ± 5.2^**^	160.2 ± 38.5	179.8 ± 49.7	47.2 ± 9.8	100.9 ± 45.9	157.6 ± 41.8^**^	28.6 ± 5.2	85.1 ± 19.9^**^
	**Total**	1000 (100)	11.2 ± 7.3	8.5 ± 5.1	161.9 ± 49.5	184.5 ± 59.8	45.3 ± 13.3	103.9 ± 48.9	184.8 ± 56.4	29.3 ± 8.1	74.4 ± 23.9
**Characteristics at the end of the study**											
**Parameters**		**Number (%)**	**HbA1c (%)**		**FBG (mg/dL)**	**TC (mg/dL)**	**HDL-C (mg/dL)**	**LDL-C (mg/dL)**	**TG (mg/dL)**	**BMI (Kg/m**^**2**^**)**	**Micro-albumin (mg/kg)**
**Gender**	**Male**	420 (45.5)	7.8 ± 2.5		139.6 ± 17.7	155.2 ± 21.3	43.6 ± 10.3	91.9 ± 18.9	148.6 ± 23.5	28.3 ± 2.7	89.5 ± 15.3
	**Female**	502 (54.5)	7.4 ± 2.2		156.8 ± 34.9	182.3 ± 35.2	45.5 ± 7.5	100.4 ± 22.5	163.5 ± 31.0	30.9 ± 5.1	71.4 ± 12.0
**Age**	**<40**	72 (7.8)	6.9 ± 1.8		135.7 ± 30.2	181.2 ± 32.4	41.2 ± 11.5	100.24 ± 19.1	161.2 ± 29.2	30.1 ± 6.8	59.1 ± 8.5
	**40–60**	338 (36.6)	7.1 ± 3.9		143.8 ± 40.1	167.3 ± 39.4	44.2 ± 10.6	93.17 ± 20.9	154.5 ± 23.7	28.2 ± 7.7	69.9 ± 9.1
	**>60**	512 (55.5)	8.0 ± 3.1		155.6 ± 22.7^**^	170.5 ± 39.8	45.2 ± 10.1	98.2 ± 37.2	157.3 ± 32.4	30.2 ± 4.4	88.4 ± 12.7^**^
	**Total**	922 (100)	7.6 ± 4.1		149.6 ± 43.4	170.0 ± 41.6	44.5 ± 12.7	96.4 ± 40.3	156.4 ± 36.5	29.4 ± 7.4	79.3 ± 17.5

*HbA1c: hemoglobin A1C, FBG: fasting blood glucose, TC: total cholesterol, HDL-C: high-density lipoprotein cholesterol, LDL-C: low-density lipoprotein cholesterol, TG: triglyceride, BMI: body mass index.

**P < 0.05 related to F test.

**Table 4:** Mean cumulative incidences of diabetes chronic complications per year

**Parameters**		**Peripheral neuropathy and diabetic foot ulcer**	**Nephropathy**	**Ophthalmic complications**	**CVD**	**Death**
**Gender**	Male	12.9 ± 1.8^**^	8.7 ± 2.0^*^	5.5 ± 0.9^*^	9.5 ± 1.5^*^	2.5 ± 0.7^*^
	Female	9.6 ± 1.8	7.6 ± 2.4	4.1 ± 0.5	8.7 ± 2.3	2.0 ± 0.6
**Age**	<40	8.1 ± 1.2	6.4 ± 0.8	3.9 ± 0.6	7.7 ± 1.1	0.5 ± 0.1
**Glycemic control level (% A1c)**	40-60	9.7 ± 1.4^*^	7.8 ± 1.3^*^	4.1 ± 0.8^*^	8.9 ± 2.1^*^	1.2 ± 0.3^*^
	>60	13.1 ± 2.3^**^	9.5 ± 0.9^**^	5.9 ± 1.4^**^	10.9 ± 2.4^**^	2.8 ± 0.1^**^
	**<7**	2.1 ± 1.1	1.4 ± 0.9	1.0 ± 0.4	1.8 ± 0.7	0.2 ± 0.1
	7-8.5	7.5 ± 2.8^*^	7.9 ± 3.1^*^	4.9 ± 2.3^*^	9.1 ± 4.1^*^	1.4 ± 0.5^*^
	>8.5	21.1 ± 7.4^**^	14.3 ± 6.7^**^	7.9 ± 3.8^**^	16.5 ± 5.6^**^	3.5 ± 1.8^*^
	**Total**	**10.9 ± 3.5**	**8.0 ± 3.1**	**4.6 ± 1.7**	**9.1 ± 3.6**	**2.3 ± 0.9**

*P < 0.05, **P < 0.01 related to F test.

**Figure 2:**
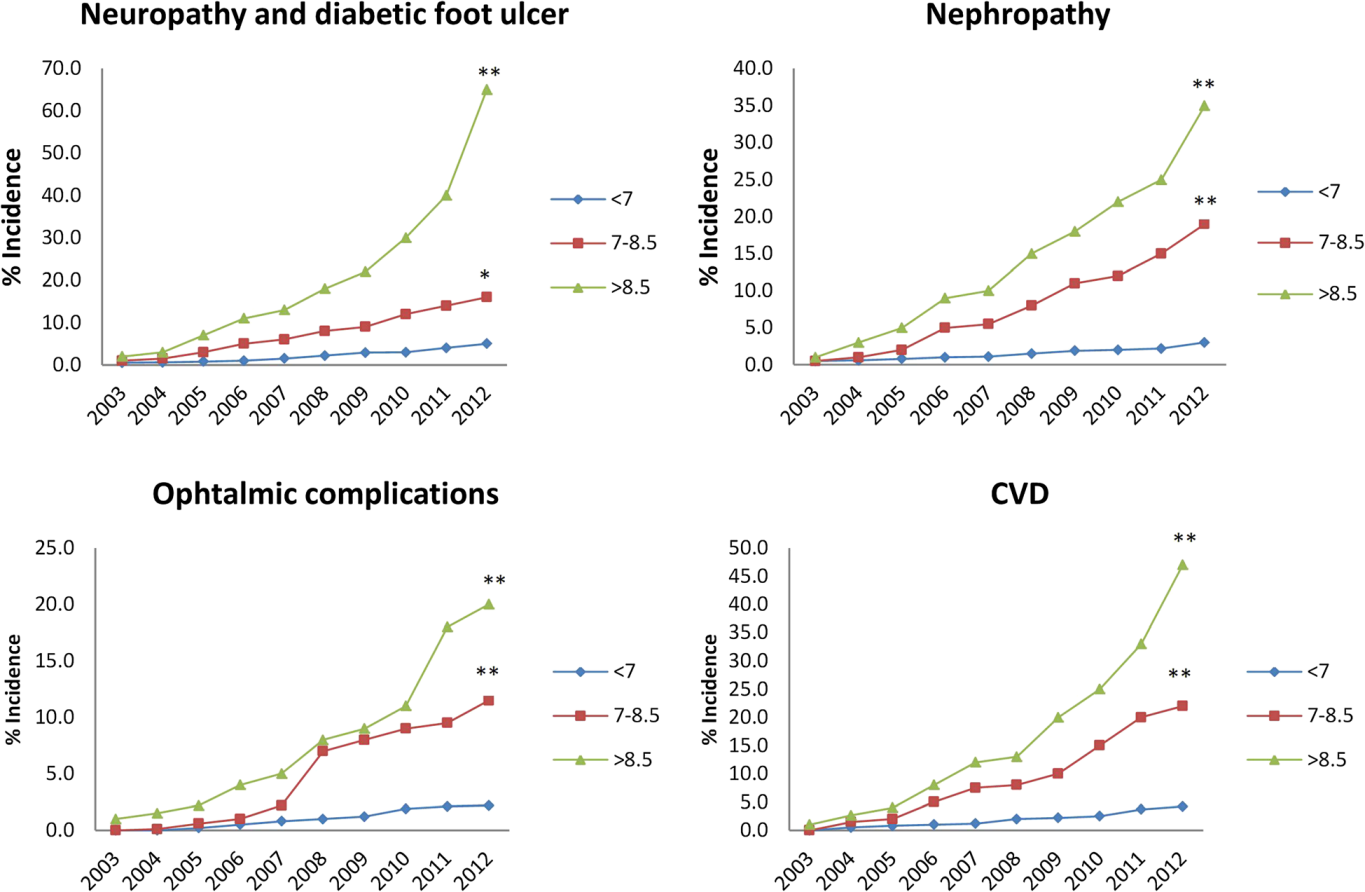
**Diabetes chronic complication incidences in categorized glycemic control (A1C %) during 2003 to 2012.** *P < 0.05, **P < 0.01 related to F test.

Stratification analysis according to the status of glycemic control is shown in Figure 2. Regarding the status of the glycemic control, 34.7% of the subjects achieved good glycemic control, while 35.0% were fairly controlled and the rest were still poorly controlled after 10 years. All people with better glycemic control had fewer incidences in diabetes chronic complications. Yearly death rate was: 0.2 ± 0.1 in people with good glycemic control; 1.4 ± 0.5 in those with fair glycemic control and 3.5 ± 1.8 in those with poor glycemic control (Table 4).

**Figure 3:**
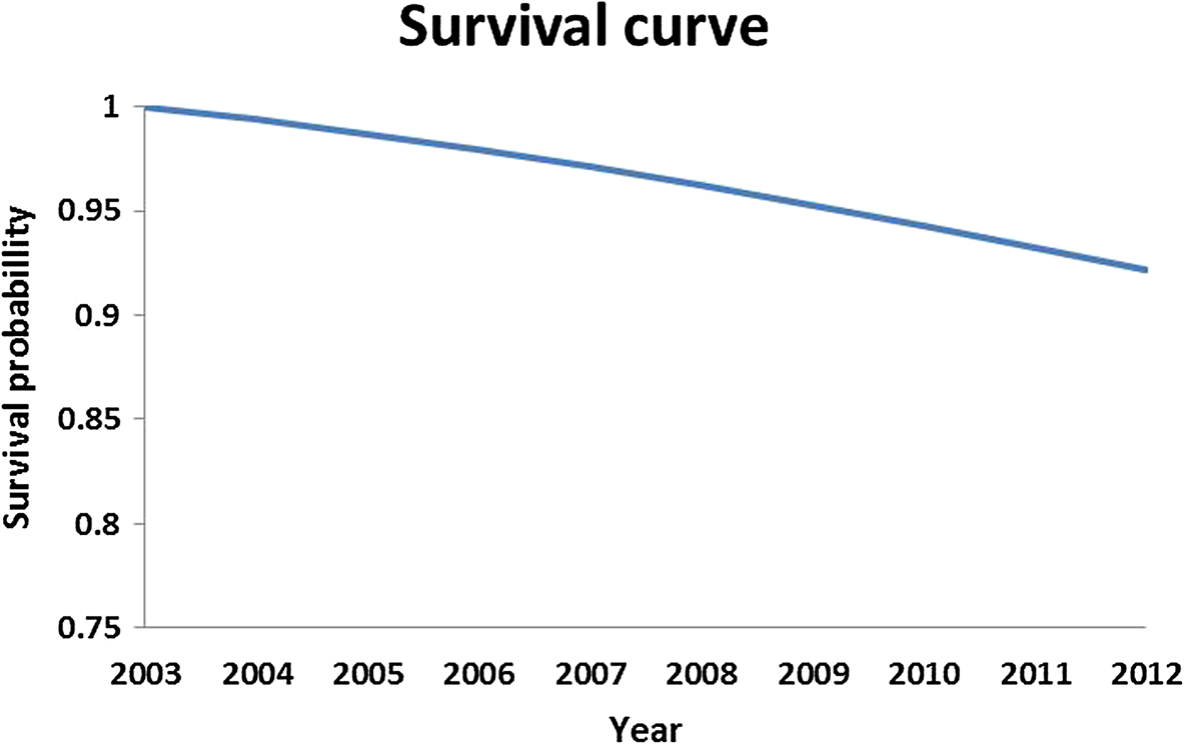
Survival curve of people with type 2 diabetes during 2003 to 2012.

In people with poor glycemic control (A1C > 8.5%) significantly higher rates of complications were seen especially in the last years (P = 0.01) (Table 4). In the last year 65%, 35%, 20% and 47% of people were suffered from peripheral neuropathy and diabetic foot ulcer, nephropathy, ophthalmic complications and CVD, respectively. The mean cumulative mortality rate was 2.3 ± 0.9% person per annum. Figure 3 shows the survival curve of people with type 2 diabetes after 10 years.

### Medication

Drug treatment was shifted from oral glucose lowering drugs (OGLDs) to insulin and OGLDs plus insulin during the 10 years. The portion of people who were treated with insulin was 5.75% at the end of the first year (1 December 2003), while it reached to 32.31% in the 10th year.

### Economic endpoints

Average direct medical cost for each chronic complication is presented with details in Table 2 while the highest expenditures were attributed to CVD. The average total cost per patient for male and female with type 2 diabetes was 1995.8 ± 104.1 and 1764.3 ± 88.14 USD, respectively in Iran. Para clinic costs included laboratory (82.2 ± 25.9), medications (225.4 ± 61.1), clinical visits (63.8 ± 22.8) and non-medical costs (22.9 ± 12.7) for the subjects. The average inpatient costs were 1599.7 ± 59.8 for males. Our cost analysis indicated that CVD and nephropathy imposed the highest expenditure in the subjects. These complications and also related costs were remarkably higher in people older than 60 years. Disability and mortality expenditures were 1050.3 ± 78.4 and 872.5 ± 49.9 among men and women, respectively. Disability and mortality costs were higher in people with 40–60 years old (1220.4 ± 63.6). The highest total cost was attributed to the age category >60 rather than the other groups (P = 0.03). Our study showed that average annually cost of each patient older than 60 years was 2277.4 ± 58.9 (Table 5). Better glycemic control was associated with less expenditures and average total cost for people with good glycemic control (A1C <7%) was 409.0 ± 43.1 while it was 3788.1 ± 110.4 for people with poor glycemic control (A1C > 8.5%) (Table 5).

**Table 5:** Yearly health care expenditure of diabetes chronic complications per patient (USD)

**Type of cost**	**Subcategory**	**Gender**		**Age (Year)**			**Glycemic control (% A1C)**		
		**Male**	**Female**	**<40**	**40-60**	**>60**	**<7**	**7-8.5**	**>8.5**
**Para-clinic**	**Laboratory**	83.3 ± 22.5	81.4 ± 19.8	74.4 ± 12.6	81.6 ± 13.3	89.9 ± 9.8	24.5 ± 8.5	78.1 ± 19.8	145.1 ± 43.2
	**Medications**	230.2 ± 57.8	221.4 ± 60.1	209.3 ± 43.2	222.4 ± 34.1	233.4 ± 23.9	74.5 ± 28.1	208.2 ± 39.7	395.6 ± 85.5
	**Clinical visits**	61.5 ± 14.2	65.8 ± 18.8	57.7 ± 12.5	63.7 ± 16.2	67.4 ± 11.2	27.6 ± 15.2	50.3 ± 21.5	110.2 ± 15.8
	**Nonmedical**	21.1 ± 7.4	24.4 ± 5.3	17.2 ± 3.9	19.5 ± 4.4	24.6 ± 5.1	8.9 ± 3.3	18.5 ± 7.8	40.5 ± 14.5
**Inpatient**	**Peripheral neuropathy and diabetic foot ulcer**	85.6 ± 4.6	71.6 ± 6.2	79.4 ± 7.1	88.6 ± 8.8	96.1 ± 6.9	21.5 ± 8.5	55.7 ± 16.8	158.9 ± 31.2
	**Nephropathy**	165.3 ± 14.4	126.1 ± 9.7	121.4 ± 15.5	133.9 ± 20.1	260.3 ± 12.5	26.8 ± 11.4	144.7 ± 55.7	265.3 ± 79.5
	**Ophthalmic complications**	84.4 ± 4.9	75.8 ± 6.3	73.2 ± 3.1	79.8 ± 11.3	88.8 ± 6.6	18.1 ± 7.9	85.9 ± 11.2	135.4 ± 21.4
	**CVD**	214.1 ± 12.8	225.3 ± 24.9	195.4 ± 19.5	212.7 ± 20.2	433.5 ± 17.3	46.6 ± 18.6	217.9 ± 32.5	397.5 ± 45.6
	**Disability and mortality**	1050.3 ± 78.4	872.5 ± 49.9	897.8 ± 37.9	1220.4 ± 63.6	983.4 ± 44.7	160.5 ± 22.6	736.9 ± 55.9	2139.6 ± 112.7
**Total cost**		1995.8 ± 104.1^*^	**1764.3 ± 88.14**	**1732.1 ± 66.3**	**2122.7 ± 51.1**	2277.4 ± 58.9^*^	**409 ± 43.1**	**1596.2 ± 47.7**	3788.1 ± 110.4^**^

*P < 0.05, **P < 0.01 related to F test.

## Discussion

The current study evaluated the incidence of micro and macrovascular complications in people with type 2 diabetes. Results of the present study indicated that diabetes chronic complications, and total healthcare costs were affected by age, gender and glycemic control as higher incidence of complications and health care expenditure were observed in the aged males (>60 years), and people with poor glycemic control (A1C >8.5%). Consistent with the Morgan study [21], the incidence of microvascular complications, particularly retinopathy and nephropathy was correlated with duration of the disease in this study. Diabetes health problems affect the economically productive people in the age range of 40–60 in the Middle East [15]. We found that middle aged people (40–60 years old) had more disability costs due to diabetes health problems which emphasis the importance of attention to people with more productivity in the society. Costs and incidence rates in men were significantly higher than women which may be because men are unwilling to seek medical advice compared to women [22]. Previous reports indicated that tight glycemic control, as close as to non-diabetic glycemic range has been effective to reduce diabetes chronic complications and overall mortality [23]. Esposti et al. in an over 2 years study showed that the mean diabetes related cost per patient was 1291.56 € in people with excellent glycemic control, 1545.99 € in those with good glycemic control, 1584.07 € in those with fair glycemic control, 1839.42 € in those with poor glycemic control, and 1894.80 € in those with very poor glycemic control [24]. Our study showed that better glycemic control could lead to less incidence of chronic diabetes complications and costs with the lowest diabetes related cost per patient in people with good control (A1C < 7%). The UK prospective diabetes study (UKPDS) reported that reduction in A1C level is likely to reduce the risk of diabetes chronic complications, with the lowest risk being in people with A1C levels in the normal range [8]. Considering the results of the current study, total per patient cost of illness for type 2 diabetes in Iran was 1914.3 ± 136.4 USD/year which is remarkably higher than the last report in 2009 (by 1707.4 USD/year) [17]. Health expenditure related to complications was 510 ± 65.3 USD, which is significantly greater than previous reports of 238.7 USD [25]. In a study conducted in Iran in 2009, it was revealed that 8.69% of total health expenditure was spent on diabetes; the estimated cost devoted was 3.78 billion USD comprising almost 2.04 billion direct (medical and non-medical) and 1.73 million indirect costs. In addition to the imposed costs of diabetes on the health system, it may reduce quality of life [17]. Therefore, a better perception of the burden may lead to the identification of more optimal treatment strategies, prevention at the individual level and prioritization of public health resources at the national level [26]. Therefore, achieving more precise data through switching to other interventions in future researches may help making more reliable decisions. Our findings demonstrated that the incidence of peripheral neuropathy and diabetic foot ulcer and CVD were higher than other diabetes-related complications.

### Peripheral neuropathy and diabetic foot ulcer

It has been discussed that 50% of people with diabetes would suffer from neuropathy during 25 years after diagnosis [27,28]. In the current study, the yearly cumulative incidence rate of peripheral neuropathy and diabetic foot ulcer was 10.9 ± 3.5% that was the highest diabetes-related complication in total study population. This incidence seems remarkably high for people with type 2 diabetes from a developing country like Iran. Abbasian et al. reported that prevalence of neuropathy in Shahroud, Iran was 77.3% [29]. Mean duration of diabetes in our population was 11.2 years. One study of north-eastern Arizona found neuropathy in 21% of people with diabetes with more than 10-year duration of disease [30]. Consistent with the other studies our study confirms the association between the incidence rate of neuropathy, age and duration of diabetes [31]. Janghorbani et al. showed the prevalence of neuropathy was higher in elderly people rather the youngers [32]. In our study, drug treatment was shifted from OGLDs to OGLDs plus insulin or insulin alone during the 10 years. The prevalence and incidence of peripheral neuropathy seemed to be higher in patients treated with insulin rather than OGLDs-treated patients [32]. A higher incidence of peripheral neuropathy among insulin-treated people could be attributed to the longer duration of diabetes, delay in insulin treatment and possible insulin neuritis at the time of neurological examination. Some studies have presented evidence of the association between poor metabolic control and increased risk or incidence of neuropathy in type 2 diabetes [33]. In our study, mean cumulative incidence of peripheral neuropathy and diabetic foot ulcer per year was: 2.1 ± 1.1 in people with good control; 7.5 ± 2.8 in those with fair control and 21.1 7.4 in those with poor control. It is found that prevalence of peripheral neuropathy in diabetes was lower in people with good glycemic control (A1C < 7%) rather than those with poor glycemic control (A1C ≥ 9%) [32,34]. In a study over 3 years of observation, the attributable cost for a 40–65 year-old with foot ulcer was 27,987 USD for 2 years after diagnosis of diabetes [34]. The cost of peripheral neuropathy and diabetic foot ulcer was 77.9 ± 8.5 in diabetic people. We demonstrated that good glycemic control could be associated with less expenditure of peripheral neuropathy and diabetic foot ulcer care. So, good glycemic control with early and comprehensive neurological investigations for peripheral neuropathy in a patient with type 2 diabetes is necessary to have better management of disease and also decrease related costs.

### Nephropathy

Nephropathy occurs in 20-40% of people 10 years after the onset of diabetes [35,36]. The incidence of nephropathy was found to be 8.0 ± 3.1% in our study, which leads to 143.9 ± 13.5 USD average cost of care in male and females. This is consistence with Amini’s report that found the incidence rate of 8.2% for diabetic nephropathy [37]. Moradi et al. stated a prevalence of 25.6% for diabetic nephropathy [38]. Other studies among Iranian people have reported the diabetic nephropathy prevalences of 13.7 and 13.9% [29,39]. In our study, a positive association was found between the incidence of nephropathy and patients’ age with the highest incidence in older people (>60 years). We found that better glycemic control (A1C < 7%) caused less incidence and also cost of nephropathy care. There are evidences that microalbuminuria is significantly associated with subsequent mortality from cardiovascular and coronary heart diseases [40]; thus, early screening for microalbuminuria and tight glycemic control, blood pressure and glomerular filtration rate in diabetic people is strongly recommended to improve the renal outcomes in order to decrease diabetic related renal diseases in the future.

### Ophthalmic complications

In the current study, ophthalmic complications had the lowest incidence rate (4.6 ± 1.7%) rather than other diabetes chronic complications. Based on our results, 78.4 ± 8.9 USD of the health expenditure was devoted to ophthalmic complications in our population. This finding is in accordance with Javanbakht’s report that ophthalmic complications had the lowest medical expenditure among type 2 diabetes chronic complications [17]. Additionally, diabetic retinopathy is a major cause of visual impairment and blindness among adults aged 20–74 years [41]. More than 60% of people with type 2 diabetes develop some degree of retinopathy after 20 years [42].

Due to changes in lifestyle and increase in ageing populations, the prevalence of ophthalmic complications is rising worldwide [43]. This is consistent with our study that people >60 years had higher incidence and cost of ophthalmic complications. This finding is supported by previous studies showing that prevalence of diabetic retinopathy is intensely related to aging and duration of the disease [21,23], though more than 90% of vision loss resulting from retinopathy can be prevented with appropriate glycemic control and ophthalmologic care [44]. Our study indicated with improvement in glycemic control (From A1C > 8.5% to A1C < 7%) a significant decrease was seen in incidence rate of retinopathy and also related health care costs in people with type 2 diabetes.

### CVD

Mortality rate among diabetic populations is mostly attributed to CVD than microvascular complications [45]. CVD is the primary cause of morbidity and mortality in diabetic people [46]. Diabetic cardiovascular complications required the highest health resource services than any other chronic complication in 2012 in the U.S. (almost 20 million USD) [47]. In the present study, it was ranked as the second complication with a mean cumulative incidence rate of 9.1 ± 3.6%, imposing approximately 214.1 ± 12.8 USD to the patient yearly. Considering the health expenditure per capita in 2010 in Iran (317 USD) [48], CVD care expenditure in diabetic people seems to be very high. Khalili et al. reported that the incidence rate of CVD among Iranian people with type 2 diabetes was 1.2% [49]. The high incidence of CVD in our study might be attributed to the fact that this study was conducted in a referral care center. Previous studies have reported an association between the degree of hyperglycemia and increased risk of myocardial infarction [50], and stroke [51]. The UKPDS showed that a reduction in the risk of myocardial infarction was associated to lower value of A1C in people with type 2 diabetes while each 1% reduction in A1C level was associated with reductions in risk of 14% for myocardial infarction [8]. In consistent with UKPDS study, our finding showed lower incidence of CVD with good glycemic control. Considering the clinical and economic impact of cardio metabolic risk factors in diabetic people, it is clear that the strategy for controlling the costs should include modification of the cardio metabolic risks in people who have already developed CVD and diabetes.

### Disability and mortality

During 10 years in the current study, the mean cumulative incidence rate of death was 2.3 ± 0.9%. Although the prevalence of diabetes is growing, data on mortality rates in affected populations are limited. A report conducted by Bertoni et al. revealed that diabetes was associated with excess mortality in elders and the rate increased with age [26]. As described in the literature, people with diabetes have lower rates of life expectancy and 85-year-old people spend only 32% of their remaining life active compared to 42% in non-diabetic people in the same age group [52]. In this study, the mortality rate was higher in men and death rates in both men and women increased significantly with age. UKPDS reported each 1% reduction in A1C was associated with 21% reductions in risk of diabetes related mortality [8]. Our results indicated that poor glycemic control resulted in higher rates of mortality (3.5 ± 1.8). However, it is clear that tight glycemic control is a fundamental factor to reduce mortality in diabetic people. The increased prevalence of diabetes among younger people suggests that it will become more common in the working-age population. It is estimated that diabetes causes a one-third reduction in earnings due to reduced productivity [53]. We showed that people in the working-age (40–60 years) have been affected more than both younger and older people; thus, the disability and mortality cost was significantly higher in this group. Tunceli showed that diabetes affected employers and society by reducing employment and also by contributing to work loss and related limitations for employed diabetic people [54]. We found that disability and mortality caused 1220.4 ± 63.6 USD costs yearly in 40-60-year age group with a significant higher expenditure in men while the lowest cost was seen in people with good glycemic control (A1C < 7%). Thus, good glycemic control as close as normal people leads to less disability and mortality in the society and have a profound impact on economic burden of diabetes.

### Medication trend

In the current study, medication was shifted from OGLDs to OGLDs plus insulin or insulin alone during the 10 years. In the first year, 12.14% of people received OGLDs plus insulin and insulin solely while this figure reached to 47.88% in the 10th year. OGLDs are preferred as the initial choice of treatment by both patients and physicians [55]. However, insulin may be considered as the first-line treatment in in lean subjects or people with severe weight loss, acute illness or systemic underlying diseases such as renal or hepatic diseases [55]. Although patient’s reluctance often delays the initiation of insulin therapy, the progressive nature of the disease and relative insulin deficiency over time will necessitate the initiation of insulin [55]. An analytical study on prescriptions in diabetic people in Iran revealed that insulin comprised 8%, 9%, 13% and 9% of all yearly prescriptions in 2006–2009, respectively. Whereas 59%, 66%, 71% and 72% of the prescription proportions were regarded to OGLDs in the same time period [56].

We found that glycemic control in older people is more difficult. On the other hand, the incidence rate of complication was higher in elderly people. Although guidelines recommend insulin therapy in uncontrolled glycemia [7], late insulin therapy causes poor glycemic control in elderly people. So, we recommended early insulinization in diabetic people to reduce diabetes-related complications. Considering increased prevalence of diabetes and the necessity of insulin therapy to reduce the risk of diabetes chronic complications it seems there is additional needs to insulin in Iran. To have a secure pharmaceutical market considering rate of disease, demands and resources [57] and also in order to prevent insulin shortage as a consequence of recent international sanctions, policy makers should pay more attention to local insulin production in Iran.

### Limitations

Although this is the only study evaluating incidence of diabetes chronic complications and related costs in the routine clinical practice during 10 years in Iran, there are some limitations. As we did not have an integral data regarding the rate of hypoglycemia in the documents, the results of direct and indirect costs may be underestimated. In addition, as the data were collected from medical records, it is possible that missing data in medical records had led to an underestimation of the costs.

## Competing interests

The authors have declared that no conflict of interest exists.

## Authors’ contributions

Designing and implementing of the study protocol, data gathering, analysis, supervising of project and manuscript drafting were performed by AF, AE, AAS, AK, FAD, MA, MEK, RA, AK and ME. All authors read and approved the final manuscript.
